# Advancing sustainable development goals through immunization: a literature review

**DOI:** 10.1186/s12992-021-00745-w

**Published:** 2021-08-26

**Authors:** Catherine Decouttere, Kim De Boeck, Nico Vandaele

**Affiliations:** KU Leuven, Access-To-Medicines research Center, Naamsestraat 69, Leuven, Belgium

**Keywords:** Immunization, Sustainable development goals, Low- and middle-income countries, Systems thinking, Health systems modeling

## Abstract

**Background:**

Immunization directly impacts health (SDG3) and brings a contribution to 14 out of the 17 Sustainable Development Goals (SDGs), such as ending poverty, reducing hunger, and reducing inequalities. Therefore, immunization is recognized to play a central role in reaching the SDGs, especially in low- and middle-income countries (LMICs). Despite continuous interventions to strengthen immunization systems and to adequately respond to emergency immunization during epidemics, the immunization-related indicators for SDG3 lag behind in sub-Saharan Africa. Especially taking into account the current Covid19 pandemic, the current performance on the connected SDGs is both a cause and a result of this.

**Methods:**

We conduct a literature review through a keyword search strategy complemented with handpicking and snowballing from earlier reviews. After title and abstract screening, we conducted a qualitative analysis of key insights and categorized them according to showing the impact of immunization on SDGs, sustainability challenges, and model-based solutions to these challenges.

**Results:**

We reveal the leveraging mechanisms triggered by immunization and position them vis-à-vis the SDGs, within the framework of Public Health and Planetary Health. Several challenges for sustainable control of vaccine-preventable diseases are identified: access to immunization services, global vaccine availability to LMICs, context-dependent vaccine effectiveness, safe and affordable vaccines, local/regional vaccine production, public-private partnerships, and immunization capacity/capability building. Model-based approaches that support SDG-promoting interventions concerning immunization systems are analyzed in light of the strategic priorities of the Immunization Agenda 2030.

**Conclusions:**

In general terms, it can be concluded that relevant future research requires (i) design for system resilience, (ii) transdisciplinary modeling, (iii) connecting interventions in immunization with SDG outcomes, (iv) designing interventions and their implementation simultaneously, (v) offering tailored solutions, and (vi) model coordination and integration of services and partnerships. The research and health community is called upon to join forces to activate existing knowledge, generate new insights and develop decision-supporting tools for Low-and Middle-Income Countries’ health authorities and communities to leverage immunization in its transformational role toward successfully meeting the SDGs in 2030.

## Background

With just one decade ahead to realize 17 ambitious but essential SDGs, most Sub-Saharan African (SSA) countries are struggling to meet and to sustain SDG3^1^ targets related to immunization: under-five mortality, elimination of vaccine-preventable diseases, and prevention of epidemics. There is a growing concern to support the transformation of immunization systems towards increased sustainability and resilience [1, 2]. The 2020 Covid-19 pandemic supports this concern clearly, as it draws global attention and funds to restoring health systems across the globe and to the development of a vaccine, in an attempt to mitigate the devastating health and economic impact of the full-blown pandemic. Health care staff in Lower and Middle-Income Countries (LMICs) need to prepare their mostly weak health systems, already overburdened by ongoing struggles with active outbreaks of infectious diseases such as Measles, Ebola, and Lassa Fever, only to name a few.

Immunization directly impacts health (SDG3) and brings a contribution to 14 out of the 17 SDGs [3]. Moreover, it has proven to be one of the most cost-effective and long-lasting health interventions [4], protecting individuals and communities both in stable times and during humanitarian crises.

Advancing both the health-related and other SDGs in LMICs through immunization requires appropriate methods and tools that can support strategic decision-making and program implementation. Furthermore, a multi-sectoral perspective within a system-based approach, that involves the relevant SDG dimensions, seems mandatory to preserve sustainability. This entails the observation that immunization is at the interface between natural and human-made systems. Although the existence of many bi-directional links between the two systems, this paper will focus on the sustainable impact of immunization on the SDGs. A core element within this system-based approach is the notion of adaptability as a means to endorse resilience.

The natural system houses pathogens (e.g., bacteria, viruses, parasites) in reservoirs such as soil, water, plants, animals, and humans. When humans are exposed to pathogens, the immune system is activated. If the activation is not effective, an infection takes place including further transmission. An infection survivor gains immunity and if enough in number, a community can develop herd immunity. Susceptibility of the population is related to the strength of the immune response against the pathogen, which is linked to age, nutrition, previous infection, and general health status. This points to the impact of immunization on other SDGs than SDG3 and vice versa.

Driven by environmental change due to natural or anthropogenic causes, such as floods, river dam constructions, conversion of forest into farmland, or climate change, pathogen ecologies adapt accordingly. This adaptation is key and gives rise to the presence of pathogens in environments where they could not flourish before. The same happens when an infected human travels or migrates to uninfected areas. A pathogen entering a new area is not recognized by the population’s naïve immune systems. Therefore it can emerge in communities and result in outbreaks, larger epidemics, or even pandemics like Covid-19.

Zoonoses are diseases that are transmissible from vertebrate animals, such as pets, livestock, or wildlife, to humans. Driven by ecological disruption and increased contact between humans and wild reservoir species, these pathogens found the opportunity to “jump the species barrier”, leading to a new human infectious disease. In specific, anthropogenic environmental disturbance, including the increased livestock population in close contact with wildlife animal populations, increased the risk of zoonotic infection from wildlife [5]. Furthermore, emerging infectious diseases are fueled by increasing population density in urban areas and the interaction between humans and wildlife, through encroachment, road building, deforestation, hunting, and global wildlife trade [5]. In addition, loss of biodiversity following anthropogenic disturbance was shown to increase the abundance of rodent-borne pathogens in central Kenya [6]. Most pandemic threats have been caused by viruses from zoonotic or vector-borne sources [7]: Ebola, SARS, MERS, H1N1 pandemic flu, and eventually also Covid-19.

These phenomena represent adaptive behavior and are clearly bidirectional in their interaction with the human-made immunization system. Concluded, there is an intimate connection between environmental, animal, and human health. The typical behavior attributed to social-ecological systems, as described by Whitmee et al. [8] applies: these systems coevolve across spatial and temporal scales, which explains endemic and emerging disease behavior, nonlinearity in disease transmission outside and during outbreaks, and scale-free phenomena such as a single adapted virus in a single infected traveler that is capable of infecting entire continents.

By providing an analysis of the existing body of research dedicated to sustainable immunization and by showing directions for future research in this field, we contribute to support the strategic priorities of the Immunization Agenda 2030 and contribute to the other SDGs.

In this paper, we discuss insights based on a literature review in which we explored (a) how immunization impacts the SDGs, (b) the factors that endanger the sustainability of immunization in LMICs (c) the research gap to enhance decision making for SDG-promoting implementations related to immunization.

## Main text

### Methods

#### Search strategy and information sources

Considering the broad array of disciplines involved, including epidemiology, system research, operations management, and anthropology, both Scopus and Pubmed databases were initially searched between January 1st 1990 and March 21, 2021. As the search term based on the SDGs needed to be expanded in order to identify papers before 2015 and papers that clearly expressed the idea behind sustainable development without mentioning the SDGs, it was replaced by variations of *sustainability* and *resilience,* which finally resulted in 3401 papers as shown in Table 1.

**Table 1:** Scopus data search

Search #	Search string
1	TS=S = (“infectious disease” OR (immunization OR immunisation) OR vaccin* OR epidemic OR outbreak) AND (system AND “Sustainable development goals”)
2	TS = (“Sustainable Development Goals”) AND (“infectious disease” OR (immunization OR immunisation) OR vaccin* OR epidemic OR outbreak)
3	TS = (humanitarian OR disaster OR emergency OR “infectious disease” OR immunization OR immunisation OR vaccin* OR epidemic OR outbreak) and (sustainab* OR resilien*) AND (system)

Similar searches were performed in Pubmed. While screening the papers based on titles and abstracts, additional papers were handpicked and found through snowballing from review papers.

#### Data extraction and synthesis

Title screening removed papers without a direct connection to the SDGs, such as theoretical topics in immunology and vaccinology, vaccine efficacy and clinical trials, technical papers on human or veterinary vaccine development, and papers related to cybersecurity.

Abstract screening mainly removed papers on livestock immunization or detailed human immunology. Similarly, papers that only briefly listed the sustainability aspect in the limitations section of their research were excluded at this point. in terms of eligibility, papers dealing with models and methods that qualify as applicable and relevant for decision-makers, implementers, and other stakeholders were included. the insights from all the resulting papers were extracted in excel for qualitative synthesis. The inclusion criteria were based on Kovacs and Moshtari [9] and Besiou, Stapleton, and Van Wassenhove [10], as shown in Table 2.

**Table 2:** Eligibility and inclusion criteria

Eligibility criteria	Inclusion criteria
• Review papers and research papers related to the sustainability of immunization operations in LMICs: long term effectiveness, equity, efficiency, resilience, adaptation • Applied research • Peer-reviewed papers and editorials	• Multidisciplinary studies describing models or methods that directly connect with SDGs • Applied to immunization or primary health care in LMICs (Besiou et al. 2011) [10] • Long term perspective, capturing existing complexity (Kovacs et al. 2019) [9] • Real context, data, involvement of stakeholders • Tool for decision-makers

#### Analysis

**Fig. 1:**
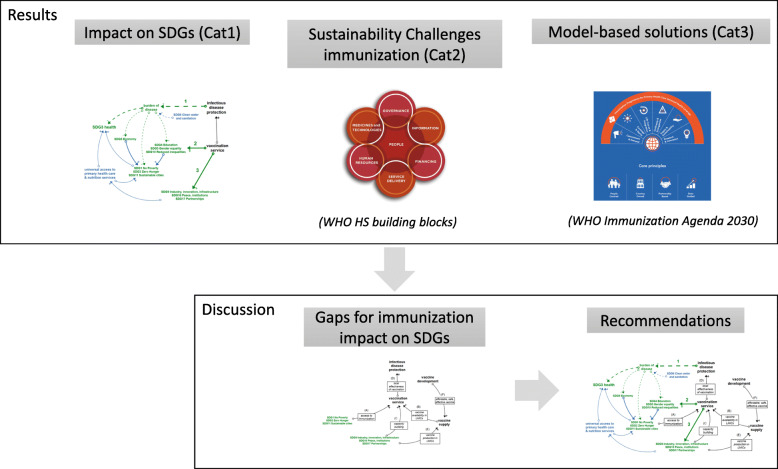
Paper structure. Paper structure combining literature analysis results with both the WHO Health System building blocks [11] and the WHO Immunization Agenda 2030 [2]

The analysis turned out the paper structure as shown in Fig. 1. All eligible and included papers, for which the numbers are listed in Table 3, were manually allocated to three categories. This has been initiated by one researcher and reviewed independently by two other researchers. A final meeting was arranged to reach a consensus.

**Table 3:** Categories of papers

Category	Immunization’s impact on SDGs (Cat1)	Sustainability challenges of immunization (Cat2)	Model-based approaches to sustainability challenges (Cat3)
Core topic	Complex relationships between SDGs and infectious disease prevention	Challenges in sustainably achieving SDG health outcomes with immunization in LMICs	Models and case-based approaches to solve challenges to achieving SDGs with immunization
Key papers (review, special issues)	[12] [8]	[13] [14]	[15–18]
Number of papers	39	205	47 (15^a^)

^a^Relevant models not fully meeting the inclusion criteria

The insights from the qualitatively analyzed papers are categorized as follows: first (Cat1), insights that explain the essential role of immunization in LMICs for reaching the SDGs; second (Cat2), insights that represent challenges concerning the sustainability of the immunization system in LMICs; and third (Cat3), insights that propose model-based approaches to these challenges. From the Cat3 papers, modeling and methodological learnings could be drawn, which support strategies for immunization system improvement and for transformation towards achieving the SDGs. By comparing the challenges (Cat2) and model-based approaches (Cat3) found in the literature, under-addressed research fields were distilled and finally, recommendations are formulated. A synopsis is given in Table 3.

Following the application of the inclusion criteria, a number of applied research papers that investigated the potential value of an intervention or optimal strategies within a single field, or in multiple fields but failing to make a connection with the SDGs, were not discussed in the Cat3 papers. However, the insights from these papers include promising elements to be leveraged by translational research in order to result in evidence-based decision support. Additionally, the interested reader is referred to De Boeck [19] for vaccine supply chain-related papers. Finally, the insights from the Cat1 papers have been used to formulate conceptual models, link the various elements and relate these elements to the SDGs, the Cat2 revealed the immunization challenges and the Cat3 allowed us to obtain an overview of model-based solutions. For each of these three steps, a focused group model building session was set up. The outcomes were iteratively validated until saturation was obtained.

## Results

The findings are structured according to the categories of Table 3. First, the impact of immunization on the SDGs is discussed. Subsequently, the challenges for the sustainability of immunization are reviewed, and finally, the model-based approaches to solve these sustainability challenges are presented.

### Impact of immunization on SDGs

**Fig. 2:**
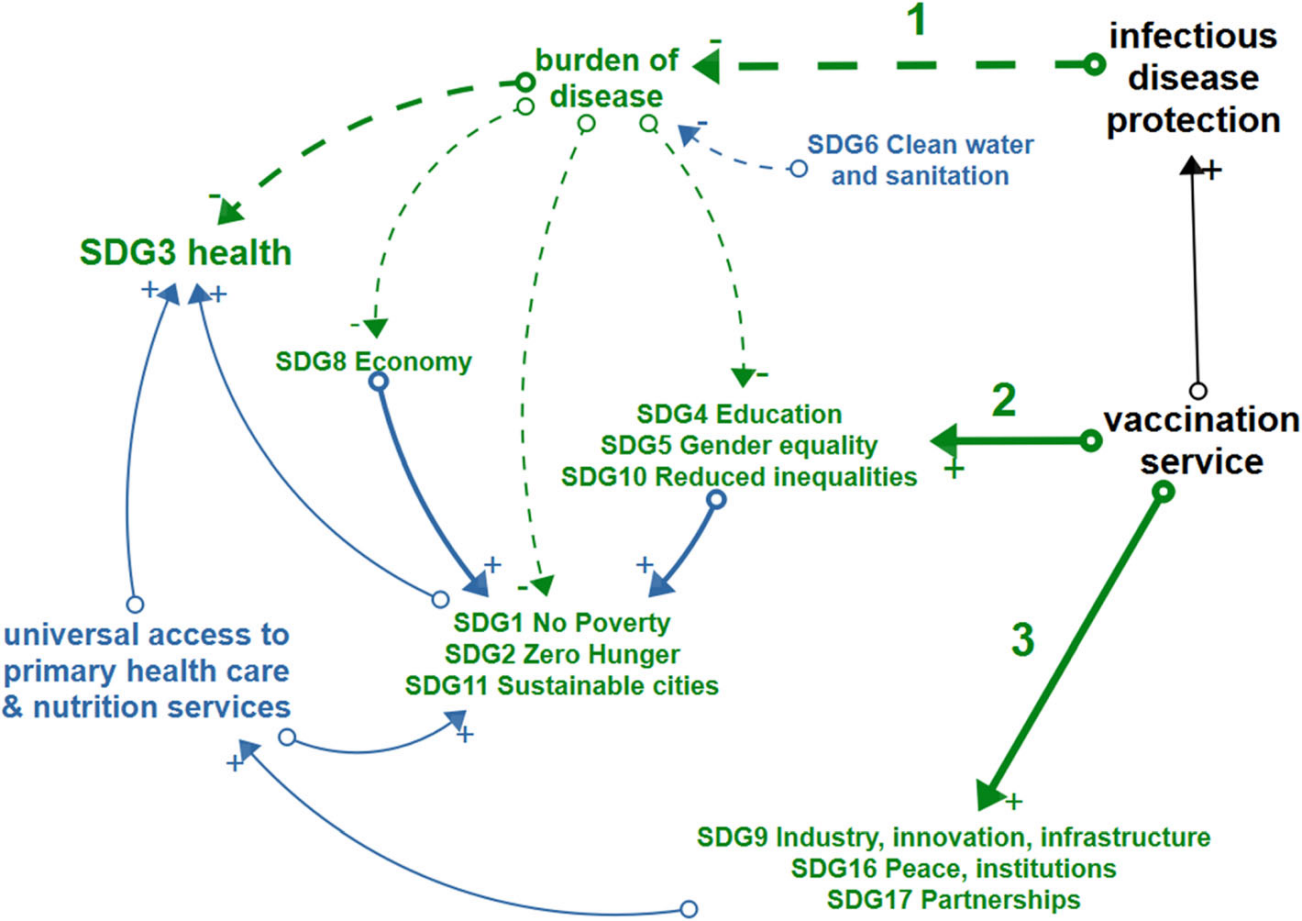
Conceptual model. Green items depict the impact of immunization on sustainable development goals which is realized through the mechanisms of (1) protecting people and communities against infectious diseases, (2) providing access to immunization services for all and (3) creating an environment for health system development. Blue arrows indicate the indirect consequences of immunization on SDGs. Black items show the overarching elements of sustainable immunization

The conceptual diagram in Fig. 2 represents the three pathways, identified from the reviewed papers, together with the ultimate impact these pathways have on the SDGs (both the pathways and the impact links are indicated in green). The first pathway (1) leads from the protection against vaccine-preventable diseases to a lower burden of disease and as such to a positive contribution to SDG3 and several other SDGs. A second pathway (2) leads from the vaccination service delivery to improved SDGs for the direct beneficiaries of immunization. A third pathway (3) also starts from vaccination service delivery and heads to SDG 17, 16, and 9, as the Expanded Program on Immunization (EPI) paves the way for partnerships in the context of health-related service delivery, that can be integrated with immunization and will lead to universal health coverage, ultimately contributing to SDG3. The indirect effects of immunization on the SDGs shown in blue, are only briefly discussed, as they lie outside the scope of this literature review.

In the following sections, the three pathways are discussed based on the Cat1 papers, referenced in Table 3, while Table 4 gives a summary of each contribution of immunization to the SDGs.

**Table 4:** Literature on the three pathways of immunization impact on SDGs

Mechanism of immunization impact on SDGs	Related SDGs	References
1. Protecting people and communities against vaccine-preventable diseases		
Avoid disease cases and epidemics Routine immunization & SIAs Surveillance and outbreak response	SDG 3 Health & Wellbeing SDG 3.2 reduce mortality SDG 3.3 end epidemics	[3, 20, 21]
Provide essential health services to everyone and limit out-of-pocket health spending	SDG 3.8 provide universal health coverage	
Emerging diseases, emergencies	SDG 3.d resilience	
Improve individuals health and increase population productivity	SDG 8 Decent work and economic growth	[22–27]
Reduction of out-of-pocket health spending pushing people into poverty	SDG 1 End poverty	
Prevention of vaccine-preventable diseases that reduce absorption of nutrients	SDG 2 Zero hunger	
Improve livelihood in urban settlements	SDG 11 Sustainable cities	
Prevent absenteeism due to disease	SDG 4 Education	
Reduce the spread of HPV, Prevent mothers to stay home taking care of sick children	SDG 5 Gender equality	
Avoiding inequity caused by epidemics	SDG 10 Reduced inequalities	
2. Providing immunization services to everyone		
Educate people on disease transmission, increase health-seeking behavior, including vaccine confidence	SDG 4 Quality education	[28, 29]
Reduce the spread of sexually transmitted infectious diseases (HIV, HPV) based on girls’ and women empowerment and easier access to immunization than to screening and treatment	SDG 5 Gender equality	
Immunization aims at reaching marginalized and vulnerable populations, reducing the health impact from their socio-eco-demographic vulnerability	SDG10 Reduced inequalities	
3. Creating the environment for health system development		
Provide the resources and conditions to build capacity for strengthening national and regional public health systems, for immunization service delivery, disease surveillance, and early diagnosis, including in conflict-affected settings	SDG 9 Industry, innovation, infrastructure SDG 16 Peace, justice, and strong institutions SDG 17 Partnerships for the goals	[30–32]
Provide incentives for public-private partnerships and capacity building for local vaccine production	SDG 9 Industry, innovation, infrastructure SDG 17 Partnerships for the goals	[33, 34]
Provides incentives for public-private partnerships for integrated health services such as family planning and nutrition services	SDG17 Partnerships for the goals	[35–38]

### Impact of protecting people and communities against vaccine-preventable diseases

One of the joint creeds of WHO and GAVI, “Immunization leads to saving lives, protecting health, and contributing to healthy and productive populations” [2, 39], summarizes the impact of protecting individuals and communities against vaccine-preventable diseases and refers primarily to SDG3 – health and well-being, but also to SDG8 - productivity. The health-related goals of the immunization system are expressed by SDG3.2 (End preventable deaths of newborns and children < 5 years of age), SDG3.3 (End epidemics of AIDS, TB, Malaria, Neglected Tropical Diseases (NTDs) and combat other communicable diseases), SDG3.8 (Universal Health Coverage (UHC)) and SDG 3.d (International Health Regulations (IHR) and increasing resilience to shocks). Immunization program outcomes are measured by immunization coverage levels and equity of immunization with respect to all vaccines in the national schedule, elimination of epidemic-prone diseases such as measles, mumps and rubella, eradication of polio, number of Human Immunodeficiency Virus (HIV) cases, new vaccines introduced against NTDs and other indicators from the Global Vaccine Action Plan (GVAP) [40] and National immunization programs [24].

Concerning SDG3.8 (UHC), the EPI programs are set up in order to immunize the target population in accordance with the GVAP targets, which propose 90% coverage rates at the national level for most vaccines, and 80% at the district level. Herd immunity, the level of immunization coverage in a population at which the chain of disease transmission is broken, is attained in most cases at around 90% immunization coverage rate (95% for measles), requiring Universal Health Coverage (UHC) and aiming at reducing under-five mortality and avoiding epidemics as stipulated by SDG3. However, it must be noted that even when national and district level GVAP targets are met, outbreaks can still occur due to under-immunization at the community level. The prevention of emerging infectious diseases (SDG3.d) is mainly focused on the development of new vaccines, and more specifically on preparing against an epidemic of disease ‘X’, for instance by the Coalition of Epidemic Preparedness Innovations (CEPI [41]).

Next to the direct goal of immunization, SDG3, the contribution to boosting the productivity of the population and economic development (SDG8) is highlighted, as a healthy workforce is critical for economic development [2, 26], which is undoubtedly undermined in case of epidemics.

On the level of households and individuals, avoiding Vaccine-Preventable Diseases (VPD) cases saves people from income loss and out-of-pocket health expenses (SDG1). In addition to malnutrition following from poverty, VPDs can lead to weakened children not taking up nutrients (SDG2), for instance in the case of cholera.

### Impact of providing access to immunization services for all

The universal access to vaccination services boosts equality (SDG5 and SDG10) and provides health education to caregivers (SDG4) together with its synergetic effects with zero hunger (SDG2), quality education (SDG4), clean water (SDG6), and climate action (SDG13) [3, 13, 29]. Furthermore, immunization’s contribution to building a productive workforce (SDG8) turns it into a core driver of country development [2, 26]. While endemic diseases by far represent the most significant burden of disease, the increasing number of outbreaks of emerging infectious diseases poses a major international concern as epidemics may rapidly spread, or even turn into a pandemic, and cause massive health, economic and emotional damage [42], illustrated by the Covid-19 pandemic.

### Impact of creating an environment for health system development

Creating an environment that is beneficial for health system development is anything but trivial. Several elements from the literature can shape this pathway. First and foremost, there is the provision of resources to build capacity for immunization service delivery, disease surveillance, and early diagnosis. This last mile capacity building is further enhanced by strengthening national and regional public health systems. In this capacity building efforts, public-private partnership plays a central role. The latter accounts both for the vaccine production [33] as well as for partnership incentives for the delivery of integrated services [32, 43], including in conflict settings where, for example, the implementation of the EPI program was found to create a “working encounter” with state and non-state actors in Myanmar which led to positive development and peacebuilding outcomes [30, 31, 35].

### Indirect impact on the SDGs

In addition to SDGs that are directly impacted by immunization through the three pathways discussed above, a number of synergetic effects exist between SDG-promoting actions, such as access to clean water (SDG6) as a complementary factor to immunization in the prevention of cholera and adequate nutrition (SDG2), which improves the immune response triggered by immunization [3, 39]. These indirect and synergetic effects are indicated in blue in Fig. 2. Moreover, the emergence and prevalence of infectious diseases is often found to be related to environmental health and animal health conditions represented by SDG13, SDG14, and SDG15 and approached as Planetary Health [8, 12, 44]. Table 5 shows an overview of hurdles and strategies, identified from the Cat1 papers, in taking these synergetic and indirect effects of immunization on the SDGs into account.

**Table 5:** Challenges in terms of indirect and synergetic effects of immunization on SDGs

Challenges	Current and suggested strategies	Papers
Transdisciplinarity, cross-sectoral collaboration and capacity building	Create a common vision and language, cross-sectoral alliances supporting OneHealth and Planetary Health	[12, 42, 44–47]
Measuring and modeling progress in multiple dimensions	Indicators for health & health environments, Composite indices	[48]
	Systems thinking to avoid trade-offs between SDGs	[49]
Holistic approach: health and well-being (SDG3) as part of broader development plan	Seek solutions closer to the source of the disease and link with SDG3 health outcomes, Ecosystem approaches. Planetary health approaches	[6, 8, 12, 44, 50–52]
	WASH interventions: provide reliable services and monitor health outcomes	[53, 54]
	Reduce vulnerability of communities. Need to quantify and understand dynamics of burden of disease from environmental change, conflict and displacement.	[44, 55]
	Lack of predictive power to accurately model human disease outcomes resulting from environmental change	[8]
Equity	Core nexus environment-economy-health, SDG integration, interdependence and implementation	[12, 26, 56, 57]
Policymaking	Considering full value of immunization. Direct and indirect effects on different SDGs, beyond cost-effectiveness	[4]
	Priority setting between long term and short term interventions	[54]
Foster resilience and adaptation, long term effects, implementation science	Long term effects, secondary effects following adaptation	[58, 59]
	Capacity building for resilience, Creating conditions that enable systems’ effectiveness	[60, 61]
	Effect of immunization on antimicrobial resistance	[62]
	Monitor sustainability of vaccine-induced immunity	[63]
	Pathogen adaptation, serotype circulation	[64, 65]
	Implementation design	[66]
Tipping points at local level: outbreaks	Surveillance at local scale, vulnerability monitoring, modeling	[67]
	Pandemic preparedness at global and local scale	[7, 68]
Complex interactions	Disease interactions, coinfections and comorbidities	[69]
Technology and innovation	Role of artificial intelligence for diagnostics	[69]

### Immunization system sustainability challenges

Immunization can only have a lasting impact on the SDGs in LMICs when immunization programs are intrinsically sustainable. Challenges observed concern the sustainability of the health outcomes reached [13], the difficulty of increasing SSA coverage levels over and above 85%, the important subnational under-immunization, and the reduced coverage rate of childhood immunization as a result of the COVID-19 pandemic [24]. Challenges for SDG3.3 are the delay in polio eradication and backward evolution in the elimination of TB, measles, and malaria, and the need for vaccine development for several NTDs such as Nipah Virus Infection and Lassa Fever, among others. In brief, at the current pace and based on the current strategies, it is expected that only SDG3.2 is likely to be achieved in SSA by 2030 [13]. Furthermore, the mechanisms that trigger epidemics and the emergence of infectious diseases related to the loss of biodiversity and climate change retrieved from the Cat1 papers will, even more, aggravate the risk of infectious diseases. In order to achieve sustainable protection against both endemic and Emerging Infectious Diseases (EIDs), there is a need for more focus on the resilience of the immunization system, than on the performance in an equilibrium state [58].

**Fig. 3:**
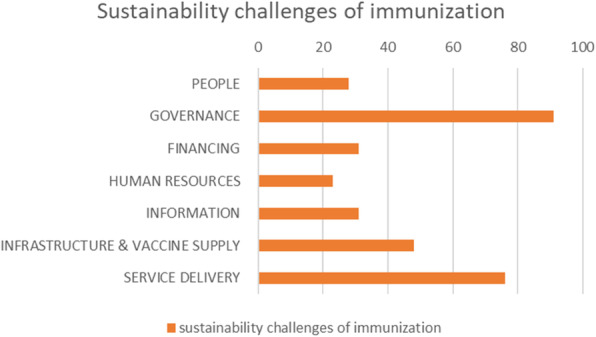
Sustainability challenges papers. Distribution of included papers, dealing with sustainability challenges of immunization in LMICs, categorized according to the WHO Health System building blocks [11]

The Cat2 papers, as described in Table 3, provide an overview of the challenges to the sustainable performance and continuation of immunization in LMICs. We found 104 papers raising issues endogenous to the national health system, 111 papers discussed topics beyond the national health system, and 9 papers dealing with issues covering both areas and counted in both, summarized in Fig. 3.

**Fig. 4:**
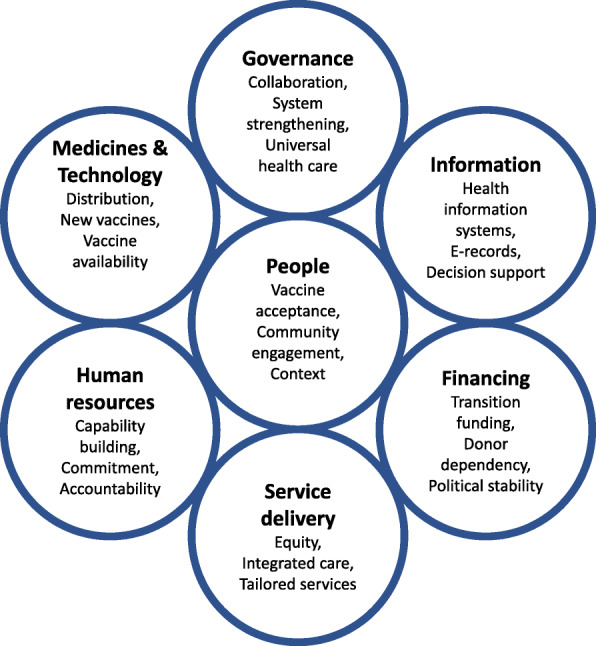
WHO Health System building blocks. Selected key sustainability challenges from the reviewed literature, according to the WHO Health System building blocks

Sustainability challenges discussed in the papers were connected to the human-centered health system building blocks as defined by the WHO [11]: *People, Governance, Financing, Human resources, Information, Medicines & Technology*, and *Service Delivery*. These are visually represented in Fig. 4, together with some selected, key illustrative sustainability challenges, derived from the extensive overview listed in Table 6.

**Table 6:** Sustainability challenges and selected references from Cat2 papers

Sustainability Challenge	References
**People**	
Vaccine acceptance	HPV vaccination: cultural acceptance, reaching girls at age of high absenteeism, education about HPV vaccines [70]
	[71–77]
Community engagement	For eradication of polio [78] Measles elimination after COVID-19 pandemic [79] [80–89]
	Impact of functioning health system and stable communities [90]
Socio-economic determinants of health	[91] Electrical infrastructure investments needed for UHC [92] [23, 26, 93]
	Migrating populations: Mixing of under-immunized with higher immunized populations in Turkey-Syria [94]
**Governance**	
Measuring performance, Data for health	[13, 69, 95–99].
Political commitment	Investments for health systems strengthening [21]
	Political endorsement and communication for vaccination. E.g., HPV vaccination [71, 90]
Need for systems thinking to connect interventions with SDG3 and other SDGs	[14, 62, 74, 75, 100–108]
New vaccine introduction and vaccination coverage	[22, 76, 109–113] NITAG decision support, absence of reliable burden of disease data [114]
Disease elimination and eradication	Behavioral challenges of strict polio strategies near eradication [59, 115, 116]
UHC and equity	Universal primary health care instead of disease-specific programs [21] [117–123]. health insurance [124]
	Need for Global Health Diplomacy to promote SDG10 as IHR were violated with COVID-19 [125]
Decision making	Decentralized decision making [13, 109, 126–130] Applying Health Technology Assessment for universal health coverage [131]
Resilience and preparedness	[132–135] Sustainability of health system through strengthening immunization, COVID-19 pandemic [136]
Collaboration	[43, 54, 82, 137–143]
	Cross-country collaboration for disease prevention, e.g. cross-border Health Initiative in Kenya and Somalia [144]
**Financing**	
Donor funding dependency	Dependency on development partner support [76, 107, 108, 145–152] In SSA the effect of ODA on under-five mortality is higher than elsewhere [153]
Transitioning out of donor funding	Transitioning out of donor funding or emergency funding [154–159]
	Difficulty of finding reliable data on budget and execution poses an issue for financial sustainability [160]
	Creating fiscal sustainability and efficiency [108, 124, 146, 148, 156, 161–164]
**Human resources**	
Capacity building, accountability, commitment	[13, 46, 82, 99, 126, 149, 163, 165–172] Technical support, open data (GIS), and supportive supervision for surveillance and disease eradication (Polio) [173] Health worker motivation and resilience [174–176]
**Information**	
Health Information Systems	[13, 72, 177–181] Home-based records must be user-centered and appropriate for local burdens of disease [182]
	Improvement of data quality is needed at the HC level related to staff but investments are usually at higher levels and in technology [183]
	Data collection, disease surveillance, Electronic health records [13, 93, 171, 184–190]
Vaccine supply chain data	Security through traceability, e.g., barcoding, GS1 [191]
Burden of disease evidence for decisions	[67, 69, 114, 123]
Data exchange systems and training	[13, 82, 121, 126, 149, 172]
**Medicines and technology**	
Vaccine distribution	Challenges of vaccine supply chains in LMICs [13, 19, 76, 107, 163, 168, 192–199]
	Technical innovations that lead to unaffordable transportation costs are not sustainable, e.g. vaccine direct delivery in Nigeria [200]
Vaccine availability to LMICs	[13, 201–209]
	Vaccine manufacturers from emerging countries. Need for good pharmacovigilance practice to build trust in vaccines [33], need for traceability, stockpiling and new packaging technologies [191, 210] Structural dependency of countries on global vaccine manufacturers, e.g. Brasil [211]
New vaccine development	[13, 63, 97, 106, 111, 212–220]
	NTDs: Need for incentives for development of vaccines for the poor [221–223]
	Packaging development for increasing coverage [224]
**Service Delivery**	
Implementation barriers and need for tailored solutions Tailored solutions	Acknowledging implementation barriers and developing strategies [25, 48, 58, 67, 79, 117–119, 225–230]
Integrated delivery platform	[70, 76, 127, 186, 189, 231–236] Implementation design equally important as intervention design [66]
	Public-private partnerships for community health [237]
Continuity	[63, 65, 175, 213, 238–241]
Resilience in service delivery	[100, 172, 242–247] Effect of conflict on performance of childhood vaccination [133] Effect of outbreaks on ongoing health prevention [248]

*ODA* Official Development Assistance

### Health system building-blocks

As an inherent feature of a complex system, the challenges discussed are often part of several building blocks. In our categorization, we opted for the dominating building block. The referenced papers, organized according to the respective building blocks, are summarized in Table 6.

#### People

Although the acknowledgment of patient-centeredness and human-centered design as an essential approach to supporting SDGs achievement has been globally confirmed, only a limited number of papers were found which explicitly discussed the challenges related to engaging and empowering people in order to achieve the SDGs or the related sustainability. While small in number (28 papers), these papers contain the main issues related to giving a voice to the “demand-side”: the target population for vaccination, or, more broadly, the individuals and communities that need protection from infectious diseases.

Other human factors that play in the supply side of vaccination, such as motivation of staff or decision making by national authorities, are either discussed under “Human resources” or “Governance.” Papers dealing with implementation challenges were categorized under “Service delivery.”

Even though nearly all papers refer to a specific geographical setting and context, generic challenges still emerged from them. First, the challenge of vaccine acceptance, which was declared by WHO as one of the ten greatest health threats to human health in 2019 [71], leads to unsustainable immunization coverage and puts a significant burden on disease elimination programs [72]. Vaccine acceptance is also an essential condition for a successful new vaccine introduction [74]. In the course of countering the current Covid19 pandemic, vaccine hesitancy in all its aspects became a considerable roadblock for immunization success [249]. Experiences with Human Papilloma Virus (HPV) introduction in Uganda, Rwanda, and Bhutan underlined the basic conditions of correct information to the population, strong political commitment, and local involvement [75]. Nevertheless, remaining cultural barriers require anthropologic research before implementation [70].

Second, a strong community engagement is required for any type of social mobilization during vaccination campaigns or routine immunization in order to create access to immunization for all. Engagement is based on connecting with the relevant community-level stakeholders and offering successful incentives. Stakeholders are traditional leaders, community health committees, and health providers, such as community health workers or “village doctors” [80, 81] Incentives are relevant when behavioral change is required for a sustainable solution. Cases of traditional nursing habits conflicting with medical insights regarding infectious disease prevention were discussed, like birth habits impacting early life bacterial exposures [89]. Further research is needed to understand the role of Community Health Workers (CHWs) in reaching the SDGs. To recover from measles resurgence after the COVID-19 pandemic, community engagement for tailored solutions must be considered [79].

Third, the challenge of universal immunization to overcome the inequality (SDG10) resulting from the socio-economic determinants of health, such as poverty and education (SDGs1–6), requires the provision of access to immunization for all [26, 91, 93]. Tied by the Environment – Economy – Health nexus [57], vicious cycles of poverty need to be broken since they lead to greater vulnerability for infectious diseases and NTDs as well as to poor access to healthcare services [23].

#### Governance

The *Governance* building-block houses decision-making entities and frameworks related to immunization target-setting, policy decision-making, and resource allocation. Moreover, the sustainability challenges found in the literature call for improved decision support based on fine-grained data and system-wide long-term models that connect interventions with SDG-level outcomes [95, 96, 103]. At the global level, it includes the immunization targets and strategies in preventing epidemic disease prevalence and outbreaks, in the broader context of Planetary Health. In addition, sustainability challenges were detected in measuring health systems’ strength for comparison between countries [95] and health systems resilience in defining and measuring performance indicators towards reaching *Grand Convergence*, and towards coping with the emerging *Double burden of disease* (the latter being the rise of non-communicable diseases in LMICs as they converge with infectious diseases as main causes of death) [13, 97–99]. At the national level, it concerns the country-specific priority setting and transformation path of health prevention and promotion as a driver for sustainable development.

A major fraction of governance-related challenges expresses the need to capture the complexity, context, and long-term perspective of the health system using systems thinking [14, 103, 104]. This is reflected in the quest for system-wide impact analysis in order to model health outcomes that result from interventions to the health system and ensuring program sustainability. Examples include the effect of changing vaccine doses per vial and vaccine thermostability [105, 106].

Similarly, political commitment (SDG16) is needed to support programs for which a direct effect is difficult to link with health outcomes, which is often the case for Health System Strengthening (HSS) programs and to synergistically integrate vertical disease-specific programs into horizontal HSS programs [21, 90]. Systems thinking is proposed as a stepping stone to dynamic modeling. Long-term system models, which reach for the 2030 SDGs and beyond, are able to show dynamic effects resulting from adaptation, such as the role of vaccines to reduce anti-microbial resistance [62], and unintended consequences, such as the behavioral reaction following mandatory immunizations [102].

Following SDG3.2, a central governance element is the continuous update of the national immunization plan and the sustainability of the Expanded Program on Immunization by taking up new vaccines and deciding on the coverage target. Challenges discussed in the papers mention that the evidence-based decision support and cost-effectiveness studies brought to the National Immunization Technical Advisory Group (NITAG), often lack the sustainability dimension and long-term or indirect effects [76, 109, 110, 112–114]. Based on these approaches, decisions on whether or not to adopt a vaccine are made without considering the full impact of the additional vaccine on the total vaccine supply chain and on the country’s epidemiology, which is impacted by all vaccines compiled in the national plan. Furthermore, the full public health value of vaccination should be measured on the population level, not only on the individual level, while taking into account the impact of non-medical elements and different SDGs, such as infrastructure works in combination with a vaccine against cholera or malaria [22, 111]. Finally, even when the local burden of disease data are not available, the NITAG needs scientifically sound decision support that captures the complex adaptive nature of the health system.

Aiming at disease elimination and polio eradication initiatives, the global coordination and national commitment to the vertical programs are of crucial importance since the last remaining disease case must be identified, and continued universal vaccination coverage is required. At the same time, disease cases are dwindling in the endgame, but disease dynamics urge for counter-intuitive strategies while the government’s commitment to the program is at risk of fading. Decision-supporting models that capture both the epidemiological dynamics and the country’s contextual landscape are needed [59, 116, 250].

Reported challenges for decision support related to UHC and equity (SDG3.8 and 3.b) were the scarcity of disaggregated data, defining differentiated approaches and strategies tailored to reach the unreached. Under-immunized populations often find themselves in humanitarian settings where case-specific immunization interventions are needed or where underlying determinants of immunization, related to other SDGs, need further investigation [21, 117–123].

In supporting the WHO’s Integrated People-Centered Health Services (IPCHS) strategy “Engaging and empowering people,” appropriate processes are needed to support decentralized decision making, enabling self-organized local solutions, and building resilience [109, 127–130]. In the case of local decision-making for enhanced EPI performance, it has been found that three conditions should be fulfilled: availability of data, understanding of the complexities in the system, and availability of decision power at the operational level [126]. Looking from the global perspective, one views self-organized local solutions as country-specific development paths to health outcomes, possibly grounded in the upscaling of proven best practices in each country [13].

In building resilience and preparedness towards disruptive events, challenges were reported regarding the relevance of cost-effectiveness-oriented optimization models for humanitarian operations and the need for incorporation in the health system of small-scale people-centered initiatives [132–134]. Adaptive behavior in a post-Ebola epidemics era gave rise to transformation strategies that need to be further implemented [135]. And of course, currently, the Covid19 pandemic showed the high need for resilient health systems in general and immunization systems in particular. It proved in a dramatic way that unpreparedness pays back not only health (SDG3) but impacts almost all SDG as mentioned earlier in our Background section.

Collaboration (SDG17) and coordination of cross-sectoral activities, between public and private partners or between nations, are needed to tackle vector-borne diseases and aim for disease eradication, integrate nutrition into the health system, foster sustainable innovation initiatives, strengthen weaker systems through regional collaboration, or apply health diplomacy to connect economic, social, and political sectors [43, 54, 82, 138–141, 143]. Understanding the interactions of these private or cross-sectoral initiatives with the health system is key. Global future-oriented health governance (SDG16) demands alignment in priorities stemming from IHR and SDGs, consultation of the global health community, understanding the relationship between health and behavior, and the role of regulation in supporting global health, as exemplified by the response to the Zika epidemic [142], or on a more permanent basis, the need for global support of synergies between horizontal and vertical programs. Global health diplomacy is proposed to avoid violation of IHR, for instance in the case of the COVID-19 pandemic [125].

#### Finance

In the context of LMIC immunization, the term “sustainability” is almost always used implicitly for “financial sustainability”. Clearly, every resource employed for immunization needs to be financed and, although the return on investment of immunization is estimated to be substantial, the funds are not available to LMIC governments to make the investments without external support. Challenges reported relate to donor dependency [76, 107, 108, 145, 147–152, 251], finding the budget from domestic sources [108, 124, 146, 148, 156, 161–164], and increased efficiency in service delivery. From the global perspective, both the transition out of GAVI support as well as the aftermath of political instability are recognized as critical milestones in the development paths of LMICs [154–159].

#### Human resources

Challenges in Human Resources were related to building capacity, accountability, and resilience. Capacity building to strengthen the scarce African healthcare human capital is needed on the level of capacity in leadership and governance, technical healthcare and supply chain skills, and in cross-sectoral disciplines in the framework of Planetary Health approaches [13, 46, 99, 163, 165, 167, 168, 170, 252]. Training programs should make a stronger link with health outcomes (SDG3) and other relevant SDGs encountered in the Planetary Health paradigm. Further challenges lie in rolling out training programs for vast numbers of local health workers and community health workers while ensuring the continuity of operations. Furthermore, synergies are expected from standardization in certificates and training programs across organizations and countries.

In the quest to obtain accountability of staff, the challenge of including health outcomes, such as under-five mortality (SDG3), in evaluation frameworks is discussed. An all-but straightforward endeavor due to the delayed effect of functioning and outcomes, and the multitude of actors in the system that play a role. Equally delicate are the challenges of installing effective and fair incentives for health care staff, without triggering unintended or unsustainable effects [166, 174–176].

A key element for health system resilience is achieved by workforce commitment and absorptive capacity, to avoid immunization service disruptions due to health workers’ frustration and subsequent strikes [174]. Therefore, an appropriate level of empowerment is needed to allow decentralized decision-making. Equally important is a balanced workload that provides buffering, and a safe environment in times of disease outbreaks involving personal risks such as during the Ebola outbreaks [172].

#### Information

Based on a review study by Kumar, routine Health Information Systems in LMICs are not utilizing their full potential of supporting the health-related SDGs (SDG3) due to Health Information System design barriers that lead to poor data quality and data use [177]. Specifically, the authors argue that user-related factors are not sufficiently embedded in the Health Information System design and propose a systems-thinking approach to cope with the Health Information System design-user reality gap [179–181]. An earlier study in Uganda pointed to the lack of standardization and strategic alignment between the health vision and the information system, on top of user-related engagement issues [178]. A principal concern in LMICs remains the considerable effort and risks involved in the transition from paper-based to electronic health registries and databases, and the consequences for improved performance in Maternal and Child Health, the cornerstone of national immunization programs. On the other hand, the availability of mobile technology has led to a proliferation of health apps resulting in 40.000 mobile health apps and hundreds of communication platforms while collaboration between health providers and the adoption of technology still experience high barriers [13].

Classified according to the twelve common applications to overcome UHC in Maternal and Child Health and the mHealth roadmap for UHC, a number of challenges for immunization were found [13, 93, 171, 184–186, 188–190, 253]. First, immunization and surveillance data collection and reporting involve identifying immunization inequity and triggering the need for enhanced interventions through immunization dashboards, such as District Health Information Software 2 also known as DHIS2. Good practices and Artificial Intelligence (AI) applications from disease-specific programs focusing on Meningitis A, Polio, and disease outbreaks in humanitarian operations, have paved the way for high-standard disease surveillance. The implementation of electronic health records is challenged with contextual factors such as power outages and usability factors. On the data usage side, electronic decision support in the form of burden of disease data to the NITAG, diagnostics tools for comorbidities of infectious diseases and non-infectious diseases, and local determinants of vaccination to drive service delivery planning are reported to be lacking. It has been found that immunization information can positively impact trust in vaccination on individual and community levels, one of the important sustainability factors. However, the information systems need to be further strengthened at this point. Health providers’ collaboration could be supported more adequately by regional and global data exchange systems and support systems for decentral decision making. In addition, provider training formats can enhance efficiency and resilience in service delivery, provided they are well accepted by the health providers and are not regarded as a threat to their expertise and value. Finally, Health Information Systems in LMICs are underutilized for supply chain management, hence the combination of the Health Information System and the Logistics Management Information System bears great potential.

#### Medicines and technology

On the level of medicines and technology, challenges in vaccine distribution are found in reaching and maintaining a sufficient level of effectiveness and efficiency in vaccine distribution and delivery, under increasingly stringent conditions resulting from population growth and the growing number of vaccines to be administered per person [13, 19, 76, 107, 163, 168, 192–195, 198, 199, 254]. Supply chain strengthening efforts to avoid out-of-stocks and to increase immunization equity experience difficulties in getting adopted beyond demonstration projects. In addition, they experience difficulties in relating to the real-life context of available data, human resources, and existing infrastructure at the lower levels of the supply chain. In specific, the introduction of new vaccines needs to be carefully considered on its full benefits and long-term sustainability, including the phase of implementation and impact on the existing immunization. A critical part of the immunization infrastructure concerns the cold chain equipment where the following sustainability challenges were detected: (i) performance of cold chain equipment ensuring the potency of the vaccines (SDG3), (ii) emissions, (iii) energy and material used during production, (iv) use, and (v) post-use stages of the cold chain equipment (SDG7, SDG13, and SDG15) depending on cooling technology applied and maintenance efforts. Future directions are the integration of vaccines with other medicines, redesign for efficiency and effectiveness, and elimination of the cold chain when vaccines would become thermostable.

Availability of vaccines to LMICs for routine or emergency situations has been troubled - on top of financing-related issues discussed earlier - by disruptions, e.g., BCG in 2014–2015 [201]. This calls for improved alignment and collaboration between immunization partners, in line with SDG17, to tackle global health risks. Similar needs occur for vaccine stockpiles against epidemic-prone diseases, which are needed even after the eradication of the disease [202]. In addition, advancements to intellectual property frameworks and optimization of regulatory pathways that can lead to a significant reduction of registration time for new vaccines for LMICs are desperately needed in order to reach UHC (SDG3) [203]. In the same context, the development and viability of domestic vaccine production appear to face a high barrier that will not be easily overcome, particularly in SSA [204–209]. Not only technological development but also trust in domestic vaccines needs to be ensured [33, 211].

New vaccine development presents a range of challenges [13, 97, 106, 111, 164, 212–220]2. First, being prepared with a vaccine to prevent outbreaks from turning into large epidemics or pandemics (SDG3.d) requires seizing the momentum for vaccine development as soon as signs of pathogenic emergence appear. However, in reality, this proved to be difficult for many reasons, not in the least due to the engagement and priority setting that needs to be set by political, scientific, and funding partners into a working coalition. Next to diseases related to visible epidemics, there is an ongoing need for vaccines against a range of endemic infectious diseases that cause a large burden of disease in LMICs amongst the most vulnerable in the population (SDG3 and SDG10), including malaria, NTDs, and HIV. Future-oriented priority setting for vaccine development, in the light of the Grand Convergence, local epidemiological needs, full public health value, and alignment with the SDGs, results in a complex decision problem. In order to safeguard the vaccine’s potency and sustainable immune response in LMICs, vaccine delivery innovations in technology or vaccination schedules are potential candidates for improvement, provided that their impact under real circumstances in LMICs can be investigated safely.

#### Service delivery

The building block of *Service Delivery* covers the tactical and operational roll-out of the programs and interventions approved and supported by *Governance*, making use of the available resources, and leading to the aspired Goals and Outcomes. As such, the sustainability challenges in immunization *Service Delivery* were grouped under the following three categories, reflecting the main goal related to the challenge: Equity, Continuity, and Resilience. Principles and methodologies from the fields of Systems Thinking, Implementation Research, and strategies from WHO’s Reaching Every District framework and IPCHS framework, were applied to operational research problems encountered in immunization service delivery. The following challenges to sustainability were noted:

Reaching equitable coverage (SDG3.2 and SDG3.8), with the aim to increase immunization above the 85% level at which it has been stagnating since 2013, is attempted by improving access through offering tailored solutions adapted to the local context. A first element that needs to be further explored is measuring vulnerability from different data sources and translating it into meaningful indicators to derive the need for immunization services, such as a fine-grained spatial vulnerability map based on a multi-dimensional poverty index, infection data, and environmental health data [67, 117, 118, 229, 230]. A second challenge concerns the design of tailored immunization delivery approaches to actually reach the under-reached, according to the accessibility of the area, population mobility, and immunization service needs [25, 48, 79, 119, 225–228]. This leads to often unique solutions regarding logistics or community engagement, which are effective in a specific context and which can be continuously improved when needed. Research papers, review papers as well as case-based research confirmed the relevance and need for an enabling environment to create tailored interventions, and to sustainably incorporate them into the health system, alongside standard interventions. A third research need lies in the configuration of an integrated delivery platform, which aims at improved people-centered care, without overstretching the health system [76, 127, 186, 231–235, 255]. Services, next to routine immunization, include nutrition, mass drug administration against NTDs, campaigns from vertical immunization programs that aim at very high coverage (Measles), or new vaccine introduction platforms. Later-age vaccinations (MMR2^2^ at 15 months) and adolescent school vaccinations (HPV^3^ at 12 years) seem to reach lower coverage levels and are more costly. It is concluded that the design of the intervention and its implementation phase are equally important and should be considered in parallel.

Second, the continuity of immunization service delivery is challenged by the increased load on the immunization system resulting from the introduction of new vaccines and population growth [63, 65, 175, 213, 238–241]. Four types of challenges were found. First, the pressure is felt at the limits of resource-related health system building blocks: the supply of vaccines and commodities, the availability of human resources, the financing from domestic and external sources, and the availability of information. These challenges were discussed in the dedicated sections above. Second, demand-side continuity factors, already discussed under *People*-building-block, which include trust in vaccination and community engagement, are threatening continuity. Third, the cost-effectiveness of operations is mentioned to endanger continuity, in particular activities for defaulter tracing, outreach, and campaigns are in need of more cost-efficient alternatives. Fourth, the sustainability of the vaccine-induced immune response must be well understood in order to plan Supplemental Immunization Activities (SIA) when needed. In addition, the surveillance of vaccine safety and adverse reactions to vaccination needs to be supported in the LMICs and must be preserved after GAVI transition. Coordination of critical immunization services, such as the implementation of switching vaccine presentations for polio eradication and rolling out SIA campaigns, add to the complexity of service delivery.

Third, resilience challenges were found in the phases of preparedness, response, and recovery in acute humanitarian emergencies [100, 172, 242–247, 256]. Preparedness is not restricted to the availability of vaccines. Equally important in times of epidemics or a pandemic is the availability of diagnostics. Experiences with preparedness for Ebola recommend mobile training for CHWs and the recognition that humanitarian organizations can contribute to developing weaker health systems in order to build resilience for acute emergencies. A number of challenges apply to the response phase, such as the competitive effect between immunization campaigns in response to disease outbreaks (e.g., meningitis A, Ebola) and routine immunization services or planned polio eradication activities. Furthermore, it was found that decision support tools for humanitarian transportation planning during emergencies should aim primarily at being implementable in acute crisis conditions, rather than aiming for optimal calculations at the expense of usability. Post-conflict and long-term conflict situations in Afghanistan and Syria showed the need for a differentiated approach to immunization to avoid dramatically low immunization coverage and loss of health gains or reemergence of conflict during the transition between emergency and development [133, 248].

## Model-based solutions to LMIC immunization sustainability challenges

Going beyond the observations of the described immunization system sustainability challenges in literature, we analyzed a third category of papers, offering model-based approaches to resolve the challenges observed in contributing to the SDGs through immunization. We specifically filtered the literature on solution approaches that comply with the inclusion criteria defined in the Methods section, and which are expected to be customizable to different LMICs and settings for re-use.

In contrast to the previous subsection, the solution approaches are classified by use of a different framework that reflects the most relevant immunization priorities in immunization for the next decade: the seven Strategic Priorities (SPs) of the WHO Immunization Agenda 2030 [2].

**Fig. 5:**
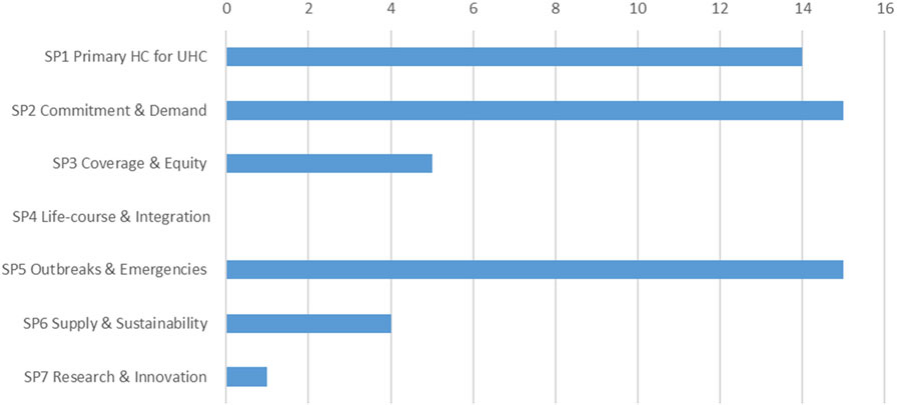
Strategic Priorities. Amount of papers with model-based solutions to Immunization system challenges classified according to the Strategic Priorities (SPs) of Immunization Agenda 2030 (version April 2020)

Based on the inclusion criteria, a total of 47 papers with models were identified, spread across the strategic priorities, as shown in Fig. 5. The main contributors were Duintjer Tebbens and Thompson [59, 116, 202, 250, 257–262], who published several papers with research mainly dedicated to polio eradication strategies and vaccine stockpiling, and Rwashana [263–266] focusing on the dynamics of the Ugandese immunization system. Compared to the entire set of papers, the purpose of the identified papers was to (i) provide the stakeholders with insight into the system’s complex behavior, (ii) deliver evidence-based decision support, (iii) demonstrate a re-usable best-practice approach, or (iv) to assess the impact of existing WHO guidelines. Methods applied in these papers include a majority of quantitative modeling approaches next to a limited number of qualitative modeling papers and papers demonstrating projects without modeling. The majority of quantitative models are based on system dynamics (SD), followed by agent-based models (ABM), analytical models, and hybrid models (the ones listed above combined with other methods such as Geographical Information System (GIS)-based spatial modeling and statistical models). The majority of qualitative models are constructed around causal-loop diagrams (CLDs), while the remaining papers apply a range of different techniques. Now we turn to a content-focused analysis of the model-based solution papers with respect to immunization sustainability.

In support of SP1, immunization programs for Primary Health Care and Universal Health Coverage, through vaccine supply chain strengthening, Lennon et al. [193] provide a generic approach for root cause analysis and institutional learning for maintenance of refrigerators, using temperature monitoring technology. Besides sustainably improved reach and efficiency of immunization programs in LMICs, local employment and ownership are positively affected, and lower emissions are expected. Through the development of high-level CLDs and System Dynamics (SD) models, Rwashana et al. [263–266] identified country-specific impacting factors, both from the supply side and the demand side, on immunization coverage and neonatal mortality in Uganda, applicable for policy design at the national level. Recently, a modeling framework for decision tools for vaccine development, aiming at universal availability has been presented [267]. Furthermore, a decision-support platform for vaccine prioritization has been put forward, for use by NITAG, including financial and other dimensions [268].

Concerning the health workforce, the recurring questions of the sustainability of performance-based financing (PBF) and the causal link between immunization coverage and PBF as a financial incentive for the immunization personnel in the health facilities were approached by qualitative systems methods by different authors. Renmans et al. [269] applied both systems’ archetypes and theory-driven hypotheses in an intervention study in Uganda, whereby the CLDs increased transparency in the complexity of interactions. Another intervention study by Alonge et al. [270] in Afghanistan resulted in a system dynamics model, while the research by Sato and Belel [271] in Nigeria was based on statistical analysis. The sustainable effect of performance-based financing on the immunization rate appeared to depend on the specific context at hand, and PBF was found to be overshadowed by the financing of the polio eradication program. This leads us to the challenge of synergistically implementing vertical disease-specific programs to contribute to HSS at the point of service delivery, which was tackled by Doherty et al. [152] by relying on qualitative appraisal methods and by Utazi et al. [272] by applying geospatial statistical modeling. On the global scale, coordination of polio eradication was supported by the System Dynamics (SD) models of Thomson et al. [257–259], which translate the intricate transmission and adaptation mechanisms of poliovirus into clear support for policymaking. As a final element in SP1, a demonstration project of a cloud-based disease surveillance system for meningitis in Burkina Faso was evaluated by Diallo et al. [185] and the sustainability of the vaccine-induced immune response for pneumococcal disease in Kenya was monitored by Ojal et al. [65] using an extended susceptible-infectious-recovered (SIR) epidemiologic model.

SP2 revolves around Commitment and Demand, referring to the continued engagement of the providers of immunization services and the beneficiaries, respectively. Commitment is supported by strengthening evidence-based decision-making with modeling tools capable of showing the long-term health impact of disease control through vaccination uptake and disease elimination and eradication through intensified vaccination strategies. In their paper, Kivuti-Bitok et al. [273] developed an SD model to support policy-making for HPV vaccination and screening in Kenya. The model runs over a time horizon up to 2050 and was designed to evolve when new information becomes available. In two of their papers on polio eradication, Duintjer Tebbens and Thompson [59, 250] focus on assisting policymaking from a long-term perspective and on acknowledging the counter-intuitive nature of the strategies proposed. Aimed at sustainably striving for UHC based on tailored solutions and empowering the district level to fulfill its role in this, Tetui et al. [274] propose an approach based on participatory action research to strengthening district health managers’ leadership capacity, as an improvement to non-participatory approaches. A comparable approach based on sense-making and discretionary power was found to sustainably support policy implementation at the Primary Health Center level in South Africa [275].

The demand for immunization relies heavily on creating sustainable public trust and vaccination confidence at the community level. To this end, Gilmore et al. [83] provided an approach based on realist evaluation to recognize the role and to support the engagement of the community health committee as a crucial stakeholder in the community-level immunization system. Sarriot et al. [276] derived a CLD for Rwanda’s integrated case management, revealing both organizational factors and context-dependent cultural motivational factors playing at national, district, and community levels in the system. In this way, they succeed in framing the role of performance-based financing in relation to political stability, sub-national program management, and utilization of services at the community level. Varghese et al. [277] also applied CLDs, among other methods applicable to *complex adaptive systems,* to reveal the basic triggers that led to stagnating vaccine acceptance in Kerala (India), resulting in dangerously low immunization coverage rates in certain districts. CLDs were also applied by Ozawa et al. [278] to explore pathways that lead to trust-building in vaccination. Through scenario analysis and mapping of health system experiences, communication, and social capital, reinforcing mechanisms and spill-over effects of distrust and the disruptive impact of the 2014 Ebola outbreak were revealed. A mathematical modeling approach in a non-LMIC context (US) was taken by Pananos et al. [279], who developed a measles outbreak prediction model based on immunization coverage and trust levels derived from social media. The approach is likely to be transferrable to LMICs. In order to activate people and communities from accepting vaccines to changing their health-related behavior Kumar et al. [89] show a community-centric design approach applied to India that aims at closing the evidence-practice gap and sustainably improving health impact. Sarriot et al. [280] investigated community learning of sustainability evaluation in a Northern Bangladesh urban health system.

Utazi et al. [272, 281] pursued SP3, Coverage and Equity, by visualizing the under-immunized population on high-resolution age-structured geographical maps, using open source data, in order to reveal inequities in vaccination coverage. This information is applicable to guide geographical prioritization and immunization strategy design for increased equity. The implementation of a sustainable immunization service delivery based on local solutions, tailored to local needs, was investigated in Ethiopia by Manyazewal [227] using a continuous quality improvement approach. Duintjer Tebbens et al. [115, 116] applied SD modeling to show the effect of polio under-vaccination on the immune response in populations of Pakistan and Afghanistan, thereby offering evidence for policymaking.

Except for the papers that provided decision support for the introduction of school-based HPV immunization targeting adolescents, no papers could be included, considering the inclusion criteria that were dedicated to supporting the delivery of SP4, Life-course and integration.

In the light of SP5, Outbreaks and Emergencies, two clusters of research resort [1]: anticipation and response to infectious disease outbreaks and [2] immunization during humanitarian crises.

First, related to anticipation and response, the prediction of disease outbreaks was tackled by Jaafar et al. [282] and Knerer et al. [283]. The authors present SD models to predict dengue outbreaks, in Malaysia and Thailand, respectively, and to evaluate combined vector-control and vaccination strategies taking into account weather conditions. In a cluster of papers, a vaccine stockpile design against a post-eradication polio outbreak was modeled by Duintjer Tebbens et al. [202] using an SD model. Later, a general vaccine stockpile design framework was proposed by Thompson et al. [260]. SD was also applied by Kalkowska et al. [262] to model polio immunity in northern Indian populations. To support outbreak response vaccination strategies during epidemics, Grais et al. [284] applied ABM to the 2003–2004 measles outbreak in Niamey. They concluded that early vaccination, and targeting a wider age range, has a larger effect with respect to managing the epidemic than putting effort and time in reaching a higher vaccination coverage in a specific age group. A similar conclusion was reached by Duijzer et al. [285] using an analytical model based on a generic case of an epidemic. In the aftermath of the 2009 H1N1 pandemic, model-based public health strategies for pandemic preparedness were investigated by Araz [286] using an SD model that considers vaccination, antiviral treatment, and non-medical interventions such as school closures. The economic impact and epidemic dynamics were evaluated on multiple criteria using Analytic Hierarchy Process along with different intervention strategies. Grefenstette et al. [287] applied ABM to census data in order to provide decision support to local authorities during epidemics. Learnings and data from the 2016 yellow fever epidemic in Angola led to the development of an adaptive vaccination strategy for Kinshasa, anticipating vaccine shortages, based on fractional dosing [288]. The 2014 outbreak in West Africa, the largest Ebola outbreak in history, exposed a painful need for a vaccine for this NTD and more adequate intervention policies. Based on data from Liberia, additional insights on missing data and human behavior during the epidemic, Pruyt et al. [289] and Auping et al. [290] developed SD models that are able to show the effect of different pro-active and reactive strategies based on available measures, such as quarantine, and -at the time- future measures, such as a vaccine. In a separate model, the authors investigate the effect of fear on health-seeking behavior and the effect of interventions on epidemic control.

Second, to support vaccination during humanitarian emergencies, research on implementable planning tools for humanitarian organizations by Gralla et al. [246] developed recommendations and concluded that heuristics-based approaches have a higher chance of being actually used compared to more complicated optimization approaches. A multi-sectoral perspective on the relations between extreme-weather-driven disasters, such as floods or droughts, and children’s health is mapped by Garcia and Sheehan [291] into a CLD which succeeds in providing an insightful overview of, on the one hand, risk factors (ecosystem and individual physical and mental health factors), and on the other hand, resilience factors (climate mitigation measures, health services, and individual coping factors). This research implies that reducing vulnerability and building individual child resilience is crucial, as immunity to VPDs under extreme-weather-driven disasters is often jeopardized.

To enable sustainable success for the immunization program, SP6 on Supply and Sustainability strives for a continued commitment to immunization materialized in vaccine supply and financial sustainability. The importance of safeguarding a national budget for health prevention and the vulnerability related to donor dependency was shown by the models of Bishai et al. [292] and Doherty et al. [152], respectively. However, broader and long-term planning models that cover multiple sectors related to different SDGs are required to design policies that encompass the EEH nexus. Similarly, these policies need to ensure fiscal sustainability needed to provide adequate health and immunization budgets.

The final strategic priority, SP7, covers Research & Innovation. A multitude of technical research papers was found, based on the search strategy, dedicated to innovations in vaccine cold chain technology, information technology, and vaccine delivery innovations. However, only the cloud-based surveillance demonstration project by Diallo et al. [185] contained a reusable demonstration project that sufficiently addressed the contributions to the SDGs, that involved human-related factors and that was context-relevant in order to be included.

## Discussion

In this Discussion section, we first examine the remaining hurdles in reaching the SDGs through immunization, along the lines and strategic priorities of the Immunization Agenda 2030. Second, we focus on hurdles specifically dealing with immunization sustainability, including the aspect of resilience and touching the current pandemic context. Third, we formulate some recommendations for future research.

### Remaining hurdles in reaching the SDGs through immunization

Figure 5 in the Results shows large literature coverage differences across the SPs. These differences are as much a reflection of the magnitude of the topics behind the SPs as it is reflecting the historic emphasis on these SPs.

Indeed, as SP1 and SP2 jointly comprise the basis of the entire immunization system, including all of the systems building blocks, by bringing them together under two strategic priorities, de facto more focus is put on their functioning as a whole towards sustainable health outcomes instead of optimizing isolated subsystems. The model-based approaches that were found within this respect show promising approaches mainly based on CLDs and SD models. In addition, where SP1 represents the organization of the supply side of the immunization system as a human-made system, SP2 concerns leadership, decision making, and engagement from both the supply as well as the demand side. Moreover, the result of all efforts depends on the immune response, which belongs to the broader natural, biological system. Modeling these different dimensions requires transdisciplinary approaches and adequate modeling techniques that can deal with highly nonlinear phenomena resulting from feedback loops and delays. CLDs and SD models were found to be successful in this endeavor. A combination of systems thinking and implementation science can be put forward to accelerate universal access to vaccines for all children in Africa, even in the Covid19 situation [20]. In a related way, implementation design and intervention design are combined through a socio-ecological model of health, applied to routine immunization in Kyrgystan [293].

SP3, coverage and equity, gained more attention in recent years as it became clear that inequitable subnational coverage led to stagnating immunization levels and below-target health outcomes in SSA. Understanding and intervening in local under-immunization often require tailored approaches that combine context-specific vulnerability and limited access to immunization. Geographical information and spatial models combined with implementation science and human-centered design were deployed here. Moreover, these factors are interconnected in the SDG context, and their root causes largely lie outside the health system. Therefore, this strategy needs further research support both from the public health and from the Planetary Health community, in order to deliver feasible solutions to actual and future inequity in immunization coverage and health.

SP4, concerning lifelong immunization, has been added very recently to the strategic priorities, as it assumes an already functioning immunization system in place, in which an individual’s immunization status can be monitored. The duration of the protective effect of immunization determines the immunization efforts needed, and only targeting children for vaccination proves to be insufficient for the lifelong protection of a population against certain VPDs. In order to evaluate the feasibility and sustainability of lifelong immunization, the impact on the entire health system, from targeting adults and the elderly through new immunization delivery platforms to the acceptance by the population, must be understood and weighed against health outcomes depending on country-specific epidemiology. Lessons learned from HPV school-based vaccination provide an initial insight and a starting point for further research. SP5, outbreaks and emergencies, received more implementable research results as this SP is located at the crossing of disease surveillance and outbreak response immunization as part of the national immunization system. At the same time, it also includes research dedicated to humanitarian operations during crises. With respect to the speed and visibility of outbreaks and epidemics, the models are often centered around a SIR logic and focus exclusively on managing the epidemic. The challenges referring to the ability of the health system to cope with the outbreak and to continue routine immunization services, i.e., the existing health system’s resilience, has not been thoroughly investigated. Its importance, however, has been underlined again during the current COVID-19 pandemic [294].

### Remaining hurdles with respect to sustainability

The challenges that came out of the literature review correspond with the Immunization Agenda 2030’s strategic priorities and core values. Under-addressed challenges include public-private partnerships, the role of sustainable innovation, cross-sectoral collaboration, and service coordination. In addition, root causes of infectious disease threat and mechanisms leading to inequitable immunization demand and access discussed in the Results section, are not directly addressed by the Immunization Agenda 2030 SPs, but appear as root causes for SP3, SP5, and SP6. Based on our review process, we did not find model-based solutions in the literature that relate interventions to these root causes with immunization and the SDGs. However, especially in the light of the COVID-19 pandemic, the need for actionable and sustainable policies to reduce the risk of future disease emergence is without a doubt.

**Fig. 6:**
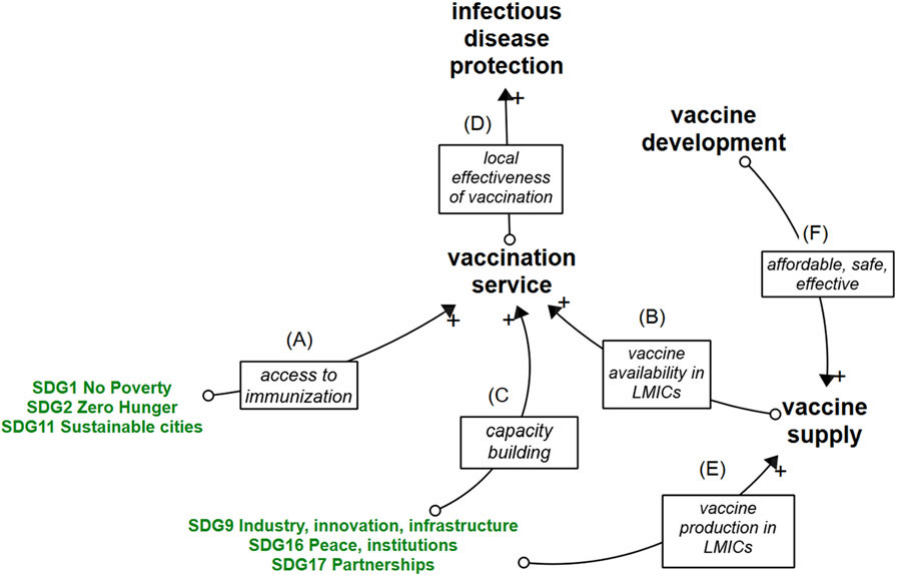
Immunization sustainability hurdles

Figure 6 represents the different stages in the immunization system and indicates challenges (A – F) when it comes to the sustainability of immunization, the prerequisite for realizing the impact on the SDGs by 2030 and beyond.

It is interesting to observe that successful vaccination service boils down to the synchronization of the three fundamental flows that meet at the vaccination service point: (A) the person to be vaccinated needs to have proper access to the vaccination service in a fair and equitable way, (B) the vaccine has to be availability under the required conditions as a consequence of well-designed and operationally effective vaccine supply policies, and (C) the presence of a professional health care worker, supported by a well-performing health system benefiting from the transdisciplinary synergies of cross-sectoral collaboration and partnerships. Even after a successful vaccination, the job is not finished. Efficient surveillance needs to be in place to assess the local effectiveness of vaccination (D) and to detect possible pathogen adaptation, leading the complex interactions with the epidemiological part of the planetary-health system, such as the fact that vaccines can lead to an adapted, vaccine-derived strain of the pathogen, e.g., Polio. Furthermore, impacting the three flow confluent point, is the need for more regional and local production in LMICs (E), a sustainable way to increase resilience when it comes to global availability and equity. Also impacting are the efforts to develop affordable, safe and effective vaccines (F) through the appropriate and collaborative R&D actions in the field of existing and emerging vaccine technology platforms combined with technological innovations across the immunization system.

Unfortunately obvious, all of these vaccination hurdles are currently illustrated by the current pandemic. We can refer to limited access to vaccination for individuals within country-wide mass vaccination campaigns, including demand-side factors such as vaccine hesitancy (A); overloaded health care systems leading to multiple capacity-related bottlenecks, including human resources (B); the global availability of vaccines, not meeting both the time and volume expectations (C); the emergence of several local Covid19 variants, showcasing the importance of surveillance of local vaccine effectiveness (D); the absence of local vaccine production in LMICs, painfully highlighting the effects of vaccine nationalism and vaccine diplomacy (E); and the increased awareness that preparedness needs to kick-off with R&D efforts, manufacturing scale-up and country readiness, long before the outbreak, as promoted by global initiatives like CEPI, COVAX, etc. (F). Needless to state that CEPI’s quest for preparedness against disease ‘X’, is more than supported by the Covid19 vaccine development efforts.

### Recommendations for future research

**Fig. 7:**
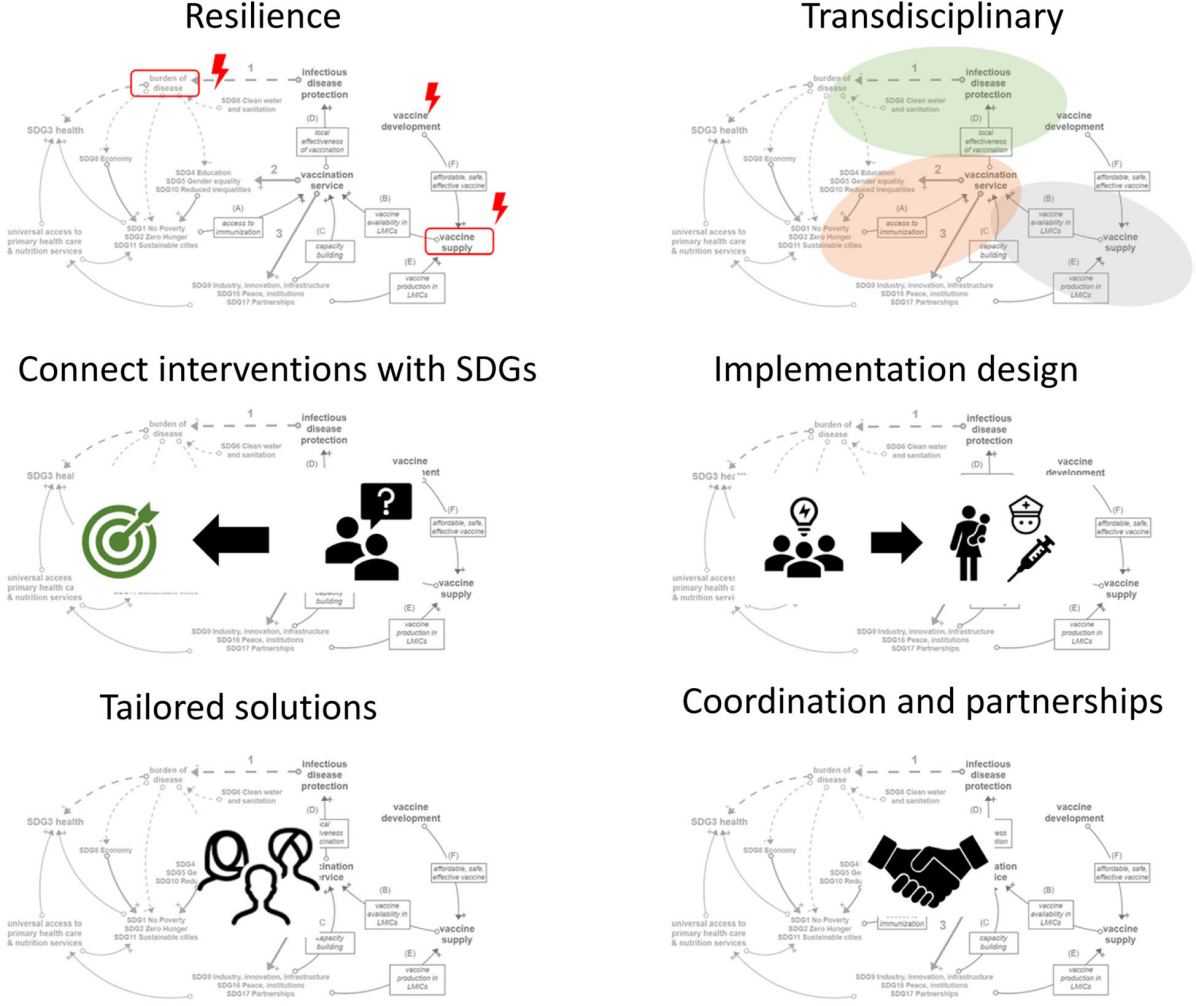
Recommendations for future research

One of the main future contributions of the academic community lies in supporting decision-making and operational management by giving insight into how immunization contributes to the SDGs, and by supporting the design and implementation of valuable interventions to improve its long-term performance towards achieving the SDGs. In line with the core values of the Immunization Agenda 2030 and the criteria for relevant research by Kovacs & Moshtari [9] and Besiou et al. [10], the following recommendations for future research were derived, also illustrated in Fig. 7.

First, in order to be sustainable, research should support the transformation of the immunization system towards stronger resilience to respond to highly nonlinear demand patterns (e.g., caused by information delays, the onset of an outbreak), adaptation mechanisms (e.g., triggered by pathogens or human behavior) and phase shifts (e.g., from routine immunization regimes to emergency response situations and back). The inherent complexity stems from the position of immunization at the interface between the natural system (the immune system) and the human-made system (the immunization system), in which the natural system ultimately sets the rules and determines the health outcomes (SDG3). Furthermore, research and humanitarian operations should support the immunization system not only to respond to but also to anticipate adaptation and increasing stress faced by the system (e.g., induced by climate change) by building resilience in stable times in between disruptive events.

Second, a transdisciplinary approach relying on systems thinking and involving both STEM (science, technology, engineering, and math) and SHAPE (social sciences, humanities, and the arts for people and the economy) experts is recommended [295] to realistically model the dynamic behavior of the immunization system, to model human behavior, to validate models, to accommodate missing data without relying on unreliable assumptions [296, 297]. This approach often includes epidemiology, human-centered design, spatial modeling using GIS, health economics, operations management, and operational research. There is a huge opportunity to activate existing research results that focused on a single subsystem, e.g., the vaccine supply chain, as pieces of information to build the comprehensive systems models that connect the subsystems. High-leverage interventions can be found at the interfaces between the subsystems.

Third, interventions should be explicitly connected to the SDGs they contribute to, with a sufficiently long time horizon, e.g., till 2030 and beyond. Interventions based on innovations should be equally sustainable. Therefore, the introduction of a new vaccine, refrigerator technology, or vaccination strategy should be measured by their impact on the SDG3-indicators and other SDGs such as Local employment (SDG8), Equity (SDG10), or Clean energy (SDG7) throughout their lifetime. In this way, the effect of the intervention on overall system performance is measured by its impact on the SDGs, and interventions leading to optimal modes of operation for only a subsystem (e.g., measured by vaccine supply chain efficiency) will be differentiated from overall optimal interventions (e.g., measured by under-five mortality, epidemic risk, equity, employment).

Fourth, the implementation design must be part of the intervention design, such that the intervention is people-centered, feasible, and adapted to the context it is intended for. The investigation of the complexity of introduction, usage, and maintenance, as well as the necessary infrastructure and skills for the intervention to work, avoids under-target outcomes from theoretically sound but infeasible interventions. For instance, human-centered design with clear stakeholder engagement is needed in order to design the type of health information system that can work in a specific community, district, or country where a specific infrastructure, leadership style, or immunization demand exists. This is in line with the five-step health system design approach of Decouttere et al. [298].

Fifth, the result could be that tailored solutions are proposed instead of generic ones in case of different needs for immunization or accessibility. In such cases, when “home-grown” solutions lead to higher engagement and resilience, they may outperform generic solutions, especially under strain conditions. Only a bottom-up research approach is able to reveal these. Therefore, research needs to investigate the appropriate setting, characterized by communities, endemic regions of a specific disease (e.g., meningitis, malaria), urban settlements, districts, and humanitarian settings. These settings do not necessarily match the administrative unit, usually delineating the modeled setting.

Sixth, research that supports the coordination of programs and partnerships, taking into account the time dimension as well as the collaboration between public and private stakeholders, has hardly been found and is definitely needed.

### Conclusion

#### Main insights

We performed a literature review covering (a) how immunization impacts the SDGs, (b) the factors that endanger the sustainability of immunization in LMICs (c) the research gap to enhance decision making for SDG-promoting implementations related to immunization.

By categorizing papers based on their SDG impact, it was confirmed that immunization can contribute to 14 of the 17 SDGs through direct and indirect mechanisms (see Background section, Fig. 1). SDG3 represents the core purpose of the immunization system, but due to the interconnectedness of the SDGs, investing in health entails increased productivity and economic development. This, in turn, reinforces the strengthening of the health system, creating a positive reinforcing relationship. In contrast, environmental health is not automatically positively impacted by both human health and economic development. For instance, population growth and anthropogenic disturbance of the natural system can trigger a change in disease ecology. This can lead to increased exposure to pathogens and infectious disease transmission. Moreover, both the exposure and the transmission occur in an inequitable fashion, further depriving already marginalized populations and thereby disproportionally increasing their vulnerability to infection. The way forward, as sketched by the SDGs, should reconcile the environment-economic-health effects without relying on trade-offs but by changing the paradigm from short-term human-focused *Public health* to SDG-supporting *Planetary health*. Such an approach takes into account the connections between the SDGs for intervention design and evaluation.

Sustainability challenges were found in all of the WHO’s Health Systems Building-Blocks, including population engagement and inequity in access to vaccination, resource limitations and workforce empowerment, vaccine supply sustainability, and governance and evidence-based decision support.

Model-based research found in literature offers implementable solutions to the sustainability challenges but needs to be further expanded in order to significantly support the WHO Immunization Agenda2030.

Recommendations for future research include a focus on resilience, transdisciplinary modeling, evaluating interventions based on the SDGs, modeling implementation along with intervention, design tailored solutions when needed, support coordination of services and partnerships.

For ease of reference, we list the basic insights from this paper in Table 7.

**Table 7:** Major insights and future research directions from the literature review

**Current state insights**	
Immunization definitely impacts many of the SDGs, both directly and indirectly. Environmental-economic-health effects induce several reinforcing loops.	
The major challenges with respect to sustainable control of vaccine preventable diseases are both supply and demand side related.	
**Demand side**	**Supply side**
Access to immunization services	Global vaccine availability
Safe and affordable vaccines	Local/regional vaccine production
Context dependent vaccine effectiveness	Public-private partnerships
	Immunization capacity/capability building
**Future directions**	
Modeling and implementation research for supporting SDG-promoting immunization system interventions in the light of the Immunization Agenda 2030.	
**Modeling**	**Implementation**
Connecting interventions in immunization with SDG outcomes	Design for system resilience
Transdisciplinary modeling	Designing interventions and their implementation simultaneously
Model coordination and synergies	Offering tailored solutions
	Integration of services and partnerships

#### Limitations

This work has several limitations when it comes to the completeness of the literature review. The keyword “sustainability” was not very effective, and even by applying exclusion and inclusion criteria, there were many more papers that could be mentioned. As the purpose was not a bibliographic review but rather an exploration of the SDG universe, the position of immunization in it, and how research can contribute to it, the paper sample was considered suitable for our purposes.

#### Relevance in times of COVID-19

As this work was written, the world witnessed the COVID-19 pandemic and its devastating effect, in line with what could be expected from the pre-2020 literature. The paths on the system map explain what is happening. However, as long as they have not led to solutions, they have no substantial impact in preparing the world for the humanitarian and economic shock wave that a pandemic causes on top of already existing epidemics, the threat of famine, and ongoing unrest. The current pandemic painfully exposes the weaknesses in existing health systems worldwide and affects the routine immunization services that will require great efforts to recover and avoid other disease outbreaks. For this reality, the research community needs to take up its role to support the transformation to more sustainable and resilient immunization systems.

## Data Availability

Not applicable.

## Abbreviations

ABM: Agent Based Modeling
CHW: Community Health Workers
CLD: Causal Loop Diagram
EID: Emerging Infectious Disease
EPI: Expanded Program on Immunization
GIS: Geographical Information System
GVAP: Global Vaccine Action program
HIV: Human Immunodeficiency Virus
HPV: Human Papilloma Virus
HS: Health System
HSS: Health System Strengthening
IHR: International Health Regulations
IPCHS: Integrated People-Centered Health Services
NITAG: National Immunization Technical Advisory Group
NTD: Neglected Tropical Disease
LMIC: Low and Middle Income Country
ODA: Official Development Assistance
PBF: Performance Based Financing
SD: System Dynamics
SHAPE: Social sciences, Humanities and the Arts for People and the Economy
SIA: Supplemental Immunization Activities
SIR: Susceptible-Infectious-Recovered
SP: Strategic Priorities
SSA: Sub Saharan Africa
STEM: Science, Technology, Engineering, and Maths
UHC: Universal Health Care
VPD: Vaccine Preventable Disease

## Footnotes

1 Ensure healthy lives and promote well-being for all at all ages (https://sustainabledevelopment.un.org/sdg3)

2 2nd dose of measles-mumps-rubella vaccine

3 Human papillomavirus
